# Concomitant sensitization to legumin, Fag e 2 and Fag e 5 predicts buckwheat allergy

**DOI:** 10.1111/cea.13068

**Published:** 2017-12-22

**Authors:** S. Geiselhart, C. Nagl, P. Dubiela, A. C. Pedersen, M. Bublin, C. Radauer, C. Bindslev‐Jensen, K. Hoffmann‐Sommergruber, C. G. Mortz

**Affiliations:** ^1^ Department of Pathophysiology and Allergy Research Medical University of Vienna Vienna Austria; ^2^ Department of Dermatology and Allergy Center Odense Research Center for Anaphylaxis (ORCA) Odense University Hospital Odense Denmark

**Keywords:** allergens, buckwheat, component‐resolved diagnosis, food allergy, IgE

## Abstract

**Background:**

Buckwheat (*Fagopyrum esculentum*) has become increasingly popular as a healthy food in Europe. However, for sensitized individuals, consumption can cause anaphylactic reactions. The aim of this study was to identify individual well‐characterized buckwheat allergens for component‐resolved diagnosis.

**Methods:**

Patients were selected by positive skin prick test to buckwheat and divided into two groups: (1) sensitized to buckwheat without clinical symptoms and (2) buckwheat allergy. Buckwheat proteins were extracted from raw buckwheat seeds, purified applying a combination of protein precipitation and chromatographic methods, and analyzed by IgE immunoblotting and ELISA.

**Results:**

Buckwheat‐allergic patients had a significantly larger median skin prick test weal diameter for buckwheat than the sensitized group and the positive control. Also, IgE immunoblotting clearly showed a distinct pattern in sera from allergic patients when compared to sensitized individuals. Several IgE‐reactive proteins were purified from crude buckwheat extract, namely legumin (Fag e 1 plus its large subunit), Fag e 2 (2S albumin), and newly identified Fag e 5 (vicilin‐like) as well as hevein‐like antimicrobial peptides, designated Fag e 4. All four allergens showed superior diagnostic precision compared to extract‐based ImmunoCAP with high sensitivity as well as high specificity.

**Conclusions:**

Patients with clinical symptoms clearly show a distinct allergen recognition pattern. We characterized a buckwheat vicilin‐like protein as a new relevant marker allergen, designated Fag e 5. Additionally, another new allergen, Fag e 4, potentially important for cross‐reactivity to latex was added to the allergen panel of buckwheat. Further, our data show that the full‐length legumin comprising both, large and small subunit should be applied for component‐resolved diagnosis. Our data indicate that concomitant sensitization to legumin, Fag e 2 and Fag e 5, predicts buckwheat allergy.

## 1 INTRODUCTION

In Asian countries, common buckwheat (*Fagopyrum esculentum*) is a popular, traditional food. Nowadays, it becomes increasingly popular also in Western countries as part of a healthy diet^1^ due to its well‐balanced amino acid composition.^2^ Buckwheat is also used as a substitute in gluten‐free food, especially for people suffering from coeliac disease.

Although the prevalence of buckwheat allergy is relatively low (0.22% in Japan, 0.11% in Korea),^3^, ^4^ it is often associated with severe anaphylaxis. Similar to peanut allergy, even small amounts can cause severe, life‐threatening reactions.^5^ This is an important issue because buckwheat is often consumed as a hidden allergen in cakes, pancakes, and pastries. In Japan, it is estimated that 2.9%‐3.4% of all reported anaphylactic events to foods are caused by buckwheat,^6^ while in Korea, buckwheat has been identified as the leading cause of food allergy.^7^ In Europe, data concerning the prevalence of buckwheat allergy are limited to date. Two Italian studies reported a sensitization prevalence of 3.6% and a prevalence of buckwheat anaphylaxis of 1%, respectively. In France, the prevalence was 4.5% of cases of food anaphylaxis.^8^, ^9^, ^10^

Currently, the widely used first‐line diagnostic approach of buckwheat allergy is skin prick test (SPT) and in vitro tests such as ImmunoCAP, to detect and quantify buckwheat‐specific IgE (sIgE). However, despite high sensitivity, the specificity of these tests are low.^11^ Thus, in many cases, food challenges are still the gold standard for a proper diagnosis.

Component‐resolved diagnosis (CRD) allows to discriminate between clinically relevant and non‐relevant sensitization for many food allergen sources (e.g. peanut, hazelnut, kiwifruit).^12^ Although buckwheat is recognized as a major food allergen source, there is limited knowledge on the causative allergens. Up to now, three important buckwheat allergens have been identified, named Fag e 1‐3. Urisu et al.^13^ characterized a protein of approximately 24 kDa with high IgE‐binding potential that was identified as the small subunit of buckwheat legumin (13S globulin) and tentatively designated Fag e 1.^14^ Predominantly severe reactions are observed in patients sensitized to a 16 kDa protein that was identified as a member of the 2S albumin family.^15^, ^16^ This 16 kDa protein was officially designated Fag e 2 (www.allergen.org). In addition, Fag e 3, a 19 kDa N‐terminal fragment of a vicilin‐like protein was identified.^17^ Most likely it originated from cleavage of a vicilin precursor protein as previously shown for a macadamia vicilin.^18^ Choi et al. concluded that sIgE to Fag e 3 correlated with true buckwheat allergy and was less prevalent in individuals only sensitized to buckwheat without clinical symptoms. Other studies also reported IgE binding to 40‐50 kDa proteins.^19^, ^20^

This study aimed to identify individual buckwheat allergens including potential marker allergens for CRD. Therefore, we evaluated and compared the allergen recognition pattern in sera from patients allergic to buckwheat and patients sensitized, but tolerant to ingestion of buckwheat, respectively. We further assessed the allergenic potential of the purified individual proteins including legumin, vicilin, and 2S albumin.

## 2 METHODS

### 2.1 Selection of patients

A total of 389 patients were tested for buckwheat sensitization by SPT at the Odense Research Center for Anaphylaxis (ORCA), Allergy Centre, Odense University Hospital, Denmark. Of these, 249 were evaluated due to suspicion of buckwheat allergy, food allergy of unknown cause or food‐dependent exercise‐induced anaphylaxis, and 140 were tested consecutively during a 4 months period. Based on a positive SPT to buckwheat, 52 patients were selected for further studies (Figure S1). The diagnosis of buckwheat allergy was verified in 21% (11/52): in 2 based on an open oral food challenge (OFC), in 2 by a double‐blind placebo‐controlled food challenge (DBPCFC), and in 7 patients by a clear‐cut case history of buckwheat allergy (intake of a product containing buckwheat eliciting anaphylaxis and other culprits excluded) combined with elevated buckwheat‐sIgE or a positive histamine release (HR) test (RefLab ApS, Copenhagen, Denmark). Patients sensitized to buckwheat but without any clinical symptoms were defined as sensitized. Sera from 34 of 52 patients were available for further studies: 27 from sensitized and 7 from allergic patients. Informed written consent was obtained from all participants, and the use of serum samples for this study was approved by the ethics committee of the Medical University of Vienna (No. 2196/2016).

Skin prick test (SPT) for food allergens was performed using fresh food (prick to prick). SPT for grass, birch, mugwort, and natural rubber latex was performed using commercial extracts (Soluprick^R^, ALK‐ABELLÓ, Hørsholm, Denmark). A positive skin reaction was defined as a mean weal diameter ≥3 mm. Specific IgE to buckwheat, other food allergens, pollen, and latex was measured using the ImmunoCAP (Thermo Fisher Scientific, Gothenburg, Sweden) with a cut‐off of 0.35 kU_A_/L. Additionally, selected sera (n = 34) were subjected to ImmunoCAP ISAC analysis (Thermo Fisher Scientific). At the time of the study, the ImmunoCAP ISAC microarray contained 112 allergens, including Fag e 2 (2S albumin) from buckwheat. IgE levels >0.3 ISU‐E were considered positive.

### 2.2 Protein extraction and purification of allergens

Protein extract used for IgE immunoblotting and for purification of allergens was prepared from raw buckwheat seeds. The seeds were frozen and ground, and proteins were extracted with 4 volumes of extraction buffer (20 mmol/L Tris‐HCl, pH 8.0, 3% polyvinyl polypyrrolidone, 10 mmol/L dithiothreitol) at 4°C overnight.

In a first step, high and low molecular mass proteins were separated by size exclusion chromatography. Further separation was performed by employing a combination of protein precipitation and ion exchange chromatography. A detailed description of the purification is given in the online repository (Figure S2, Supplementary Methods).

### 2.3 SDS‐PAGE and immunoblotting

Protein extracts from buckwheat as well as purified proteins were separated by SDS‐PAGE (12% or 18%) and blotted onto nitrocellulose membranes (0.2 μm). Membranes were blocked and incubated overnight with patients’ sera (diluted 1:5) at 4°C. Bound IgE was detected by incubation with ^125^I‐labelled anti‐human IgE (BSM Diagnostica, Vienna, Austria) diluted 1:15 overnight at room temperature or by incubation with HRP‐labelled anti‐human IgE (KPL, Gaithersburg, USA) diluted 1:5000 overnight at room temperature and chemiluminescent detection.

### 2.4 IgE ELISA

Microtitre plates (Nunc, Roskilde, Denmark) were coated with 3 μg buckwheat extract or 0.2 μg purified protein (legumin, Fag e 2, Fag e 4, Fag e 5) per well and blocked by incubation in Tris‐buffered saline containing 0.5% (v/v) Tween 20 (TBST) and 3% (w/v) BSA. Sera from sensitized individuals, allergic patients, and non‐allergic control subjects diluted 1:10 were applied onto the plates overnight at 4°C. Bound IgE was detected by incubation with a 1:1000 diluted alkaline phosphatase‐conjugated mouse anti‐human IgE antibody (BD BioSciences, Heidelberg, Germany) for 2 hours at room temperature, and colour development was performed using disodium p‐nitrophenyl phosphate substrate tablets. OD was measured at 405 nm, and the mean value of the negative controls (healthy donors) plus 3× SD was used as the threshold. All sera were tested in duplicates.

### 2.5 Statistics

Results are given as medians and ranges. Statistical analyses were performed using the Mann‐Whitney test for differences between independent samples and Chi square for differences between groups. *P*‐values below .05 were considered significant. Analyses were performed with GraphPad Prism (GraphPad Software, La Jolla, USA) and STATA12 SE (Stata Corporation, College Station, TX, USA).

## 3 RESULTS

### 3.1 Clinical data

Fifty‐two patients, 34 female and 18 male (median age 41, range 6‐71 years), with a positive SPT for buckwheat were included in this study. The patients were divided into two subgroups (Figure S1); the allergic group included 11 patients, diagnosed with buckwheat allergy (median age 52, range 34‐67 years), and the sensitized group included 41 subjects who were sensitized without clinical symptoms upon oral intake of buckwheat (median age 33, range 6‐71 years). All buckwheat‐allergic patients had experienced a severe allergic reaction to food containing buckwheat such a pancakes, blinis, or cake. According to Sampson's severity score,^21^ all patients in this study reacted with anaphylaxis grade 2‐5 (Table S1). Among patients from the buckwheat‐allergic group, eight patients were tested for buckwheat‐sIgE and all were positive. From the SPT‐sensitized subgroup, 18 were tested for buckwheat‐sIgE and ten were positive (55.5%). Of the 10 in the sensitized subgroup, five had a negative food challenge, one patient had consumed buckwheat at home but without symptoms, and four had no clinical suspicion of buckwheat allergy (Figure S1). Median sIgE for SPT‐sensitized and allergic patients was 0.65 kU_A_/L (range: <0.35‐14.2 kU_A_/L) and 4.7 kU_A_/L (range: 1.9‐36.9 kU_A_/L), respectively. There was a significant difference between the groups (*P* < .002). Buckwheat‐allergic patients had a significantly larger median SPT weal diameter for buckwheat than the sensitized group (*P* < .0001) (median: 10.0 mm vs 4.5 mm). The median weal diameter in allergic individuals was significantly larger than the diameter of the positive control (*P* < .0003) (median: 10.0 mm vs 6.0 mm), whereas the median diameter of the sensitized group was significantly smaller than that of the controls (*P* < .0001) (median: 4.5 mm vs 6.5 mm). Data are summarized in Table 1.

**Table 1:** Characteristics of the study population

	BW‐sensitized	BW‐allergic
No. of patients	41	11
Age		
Mean	33	52
Range	6‐71	34‐67
Sex		
Male, no.	18	0
Female, no.	23	11
Symptoms to BW		
Skin	0	10
GI tract	0	6
Respiratory tract	0	8
Cardiovascular	0	3
Neurological	0	2
Food challenge		
Positive	0	4
Negative	6	0
Not done	35	7
SPT for buckwheat (median weal diameter, mm)	4.5	10
BW‐specific IgE		
Positive	10	8
Negative	8	0
Not done	23	3
Food sensitization (SPT)	No. pos/No. tested	No. pos/No. tested
Cereal grains	20/28	0/7
Hazelnut	26/37	2/8
Peanut	14/37	0/8
Poppy seed	15/36	0/6
Sesame	19/36	0/6
Soy	17/37	0/8
Wheat	28/41	0/9
Sensitization to inhalant allergen sources (SPT)		
Grass	28/40	4/10
Birch	18/38	1/10
Mugwort	15/38	2/9
Sensitization to latex (SPT)	4/32	0/8

BW, buckwheat; GI, gastro intestinal; SPT, skin prick test.

Cosensitizations to pollen, foods, and latex were tested by SPT (Table S2). Among the buckwheat‐sensitized, 80% were sensitized to one or more pollen compared to 50% in the buckwheat‐allergic group (*P* = .05). In the sensitized group, 20% reacted to all 3 pollen compared to 0% in the buckwheat‐allergic group. Buckwheat‐sensitized patients were sensitized to hazelnut (70%), cereal grains (71%), wheat (68%), sesame (53%), soy (46%), poppy seed (42%), peanut (38%), and latex (12.5%). In contrast, none of the buckwheat‐allergic patients were sensitized to foods or latex.

Table S3 summarizes IgE cosensitizations among buckwheat‐allergic and buckwheat‐sensitized patients. All buckwheat‐sensitized patients positive for sIgE were tested positive for peanut‐specific IgE. Of these, two were diagnosed with peanut allergy also having the highest buckwheat‐specific IgE in the buckwheat‐sensitized group, 14.2 and 6 kUA/L, respectively. Nine of 10 and 6 of 9 buckwheat‐sensitized IgE positive patients were also cosensitized to grass pollen and birch pollen, respectively. Four of 11 and 0 of 10 allergic patients were cosensitized to grass pollen and birch pollen, respectively (Table S3).

To obtain a better overview of cosensitization patterns of the two groups, we analyzed 34 of the buckwheat samples (27 sensitized and 7 allergic), where serum was available, using ImmunoCAP ISAC. However, the only buckwheat component on the ISAC was Fag e 2. The analysis revealed that only three of seven buckwheat‐allergic patients’ sera and 1 of 27 of sensitized displayed Fag e 2‐specific IgE (A3, A4, and A11). Interestingly, these sera were tested negative for all other components (112 in total). Four of seven (A1, A6, A7, and A9) did not show any positive reaction on the chip. In the buckwheat‐sensitized group, 10 of 27 did not recognize any allergen. The results are summarized in Table S4.

### 3.2 IgE immunoblotting with buckwheat extract clearly shows a distinct pattern in allergic patients

**Figure 1:**
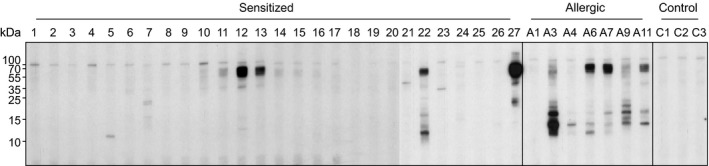
IgE immunoblot of buckwheat extract with sera from buckwheat‐sensitized (S1‐S27) and buckwheat‐allergic (A1‐A11) patients. Sera from healthy donors served as controls (C1‐C3). Molecular masses (kDa) are indicated on the left

IgE allergen recognition patterns were analysed using sera from 27 sensitized and 7 allergic patients (Figure 1). At non‐reducing conditions, several allergens ranging from 10 to 70 kDa were identified. The highest binding prevalence was observed in the range of 55‐70, 16, and 12 kDa. Sera from patients with clinical symptoms showed different IgE recognition patterns when compared to the buckwheat‐sensitized group. Six of 7 sera from buckwheat‐allergic patients contained buckwheat‐sIgE, whereas in the sensitized group only 4 of 27 showed IgE binding, predominantly to the 55‐70 kDa protein group.

### 3.3 Purification and identification of several important buckwheat allergens

**Figure 2:**
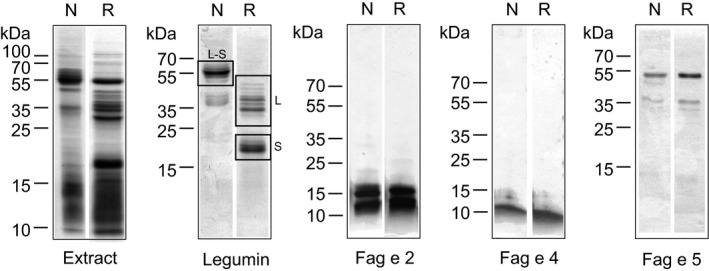
Coomassie‐stained SDS‐PAGE (12% or 18%) of crude buckwheat extract and the different purified proteins at non‐reducing (N) and reducing (R) conditions. Legumin (L‐S; disulphide‐linked large and small subunit); large subunit (L) of legumin; small subunit (S; Fag e 1) of legumin. Molecular masses (kDa) are indicated on the left

To identify and characterize the IgE‐binding proteins, we purified the components using a combination of precipitation and chromatographic methods. The purified proteins were identified by N‐terminal sequencing and/or tandem mass spectrometry or confirmed by MALDI‐TOF mass spectrometry (Figure S3A‐D). Figure 2 shows all purified components at reducing and non‐reducing conditions. The most abundant component was legumin (L‐S, disulphide‐linked small and large subunit; 55‐70 kDa), which after reduction was separated into a small (S; Fag e 1; 18‐22 kDa) and a large subunit (L; 33‐38 kDa), providing the typical pattern of legumins.^22^ Immunoblots using purified legumin were performed at non‐reducing and reducing conditions (Figure S4). Binding of sIgE to the native protein (L‐S) was observed when testing sera from buckwheat‐allergic patients (5 of 7 sera). However, upon reduction, only 2 of 7 sera recognized the small subunit (Figure S4). In addition, sera from 2 healthy donors as well as 3 from sensitized individuals also displayed IgE binding to the small subunit.

An additional purified protein separated into two major bands of about 55 kDa and ~35 kDa exhibiting only small changes in mobility after reduction. Mass spectrometric analysis identified it as a member of the vicilin family of allergens (7S globulin) and has been designated Fag e 5 by the IUIS allergen nomenclature subcommittee. However, the sequence of the purified protein did not contain the N‐terminal fragment that has been identified previously as IgE‐binding protein Fag e 3.

Furthermore, small IgE‐binding proteins of low abundance were identified as hevein‐like antimicrobial peptides 1 and 2 (AMP1/2) only differing in one amino acid residue^23^ and have been designated Fag e 4 by the IUIS allergen nomenclature subcommittee. Finally, we purified the 16 kDa component, the 2S albumin Fag e 2.

### 3.4 Application of single well‐characterized allergens

**Figure 3:**
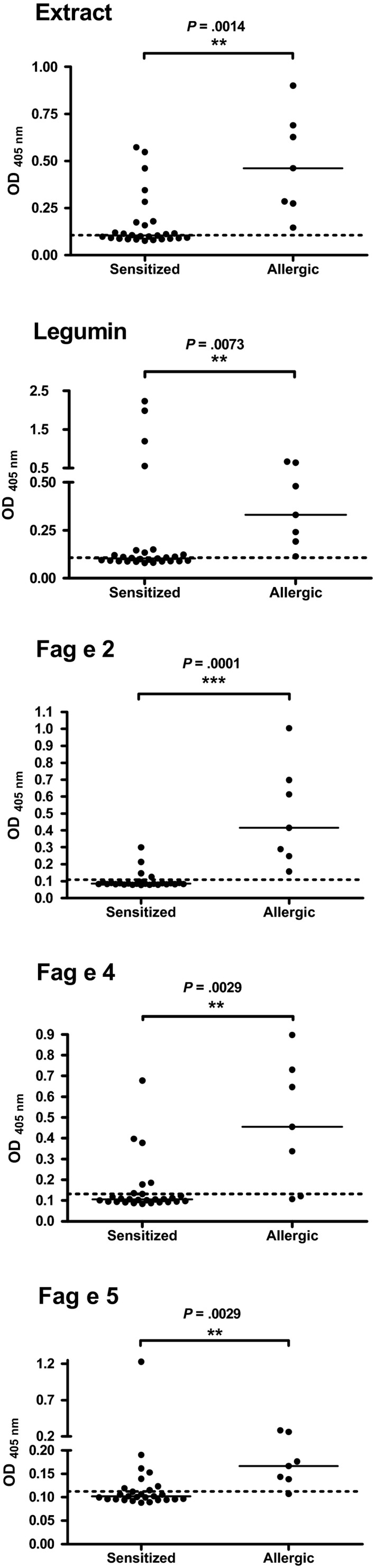
Results of ELISA comparing the two groups of patients, buckwheat‐sensitized but tolerant vs allergic, for buckwheat extract and the individual purified components. Black lines indicate median values of the respective groups; dashed lines indicate cut‐off levels (healthy donors)

**Figure 4:**
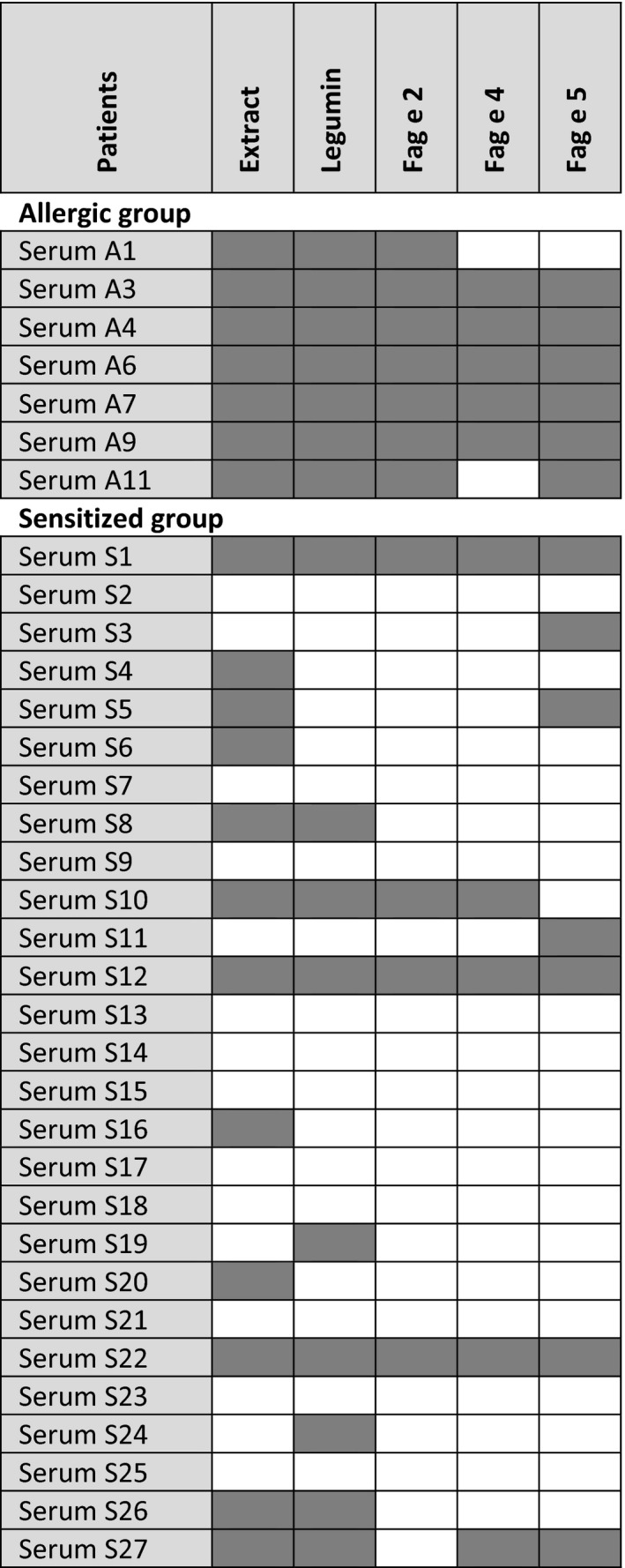
IgE reactivity profiles to buckwheat extract and individual allergens in buckwheat‐allergic and buckwheat‐sensitized patients

Next, sera from buckwheat‐sensitized and buckwheat‐allergic patients were analyzed for their recognition of self‐prepared buckwheat extract and purified legumin, Fag e 2, Fag e 4, and Fag e 5 by ELISA (Figure 3). Sera from buckwheat‐allergic patients contained significantly higher levels of sIgE to buckwheat extract (median: 0.10 vs 0.46; *P* = .0014), legumin (L‐S) (median: 0.10 vs 0.33; *P* = .0073), Fag e 2 (median: 0.08 vs 0.42; *P* = .0001), Fag e 4 (median: 0.11 vs 0.46; *P* = .0029), and Fag e 5 (mature protein without the N‐terminal fragment Fag e 3) (median: 0.10 vs 0.17; *P* = .0029). All sera from buckwheat‐allergic patients reacted to purified legumin (L‐S; 55‐70 kDa). Interestingly, four of the sensitized group showed extremely high IgE levels, even higher than sera from the allergic group. All sera from buckwheat‐allergic patients displayed anti‐Fag e 2‐specific IgE. Within the sensitized group, only 4 of 27 had Fag e 2‐sIgE. Six of 7 allergic patients’ sera showed a strong reaction to Fag e 5. The newly identified allergen Fag e 4 was recognized by 5 of 7 sera from buckwheat‐allergic patients. Figure 4 gives an overview on the individual IgE reactivity profiles of patients with buckwheat allergy and patients sensitized to buckwheat.

Finally, the sensitivities and specificities of ImmunoCAP, ISAC, and ELISA‐tests were calculated and are shown in Table 2. Although ImmunoCAP analysis showed a sensitivity of 100%, the specificity was rather low (44%). Fag e 2 on the ISAC chip had a sensitivity of only 43% and a specificity of 96%. Analysis performed for the different components, namely legumin, Fag e 2, Fag e 4, and Fag e 5, revealed sensitivities of 100%, 100%, 71%, and 86%, and specificities of 67%, 85%, 81%, and 74%, respectively.

**Table 2:** Diagnostic performance of the different methods/components

	Sensitivity (%)	Specificity (%)	BW‐allergic No. pos/No. neg	BW‐sensitized No. pos/No. neg
ImmunoCAP: Extract	100	44	7/0	8/10
ISAC: Fag e 2	43	96	3/4	1/26
ELISA: Extract	100	56	7/0	12/15
BW‐legumin	100	67	7/0	9/18
Fag e 2	100	85	7/0	4/23
Fag e 4	71	81	5/2	5/22
Fag e 5	86	74	6/1	7/20

BW, buckwheat; ISAC, immune solid‐phase allergen chip.

## 4 DISCUSSION

In this study, we examined the allergen recognition patterns in a cohort of Danish patients clinically allergic versus sensitized to buckwheat. We purified individual buckwheat allergens: legumin, Fag e 2 (2S albumin), and the newly identified allergens Fag e 4 (antimicrobial peptides belonging to the hevein family) and Fag e 5 (vicilin‐like), respectively. The performance of the individual allergens was compared to commercially available tests (ImmunoCAP, ImmunoCAP ISAC). Moreover, IgE immunoblotting was performed using the self‐prepared buckwheat extract. Within our cohort, patients with clinical symptoms upon buckwheat consumption clearly showed a distinct allergen recognition pattern when compared to sensitized but tolerant individuals. This is consistent with previous studies showing that immunoblotting profiles differed between the groups of patients.^19^, ^24^ However, these studies were solely based on the molecular masses and intensities of the IgE‐binding proteins present in an extract and lacked further information about the involved allergenic proteins.

We showed that full‐length buckwheat legumin (L‐S; 55‐70 kDa) from the seed storage protein family did not only provide a strong signal on the immunoblot, but also bound significantly more sIgE in sera from allergic patients as compared to the control group. Previous reports described the small subunit of the legumin (BW24KD)^13^ as a major allergen, also designated Fag e 1.^14^ In our study, full‐length legumin (L‐S; 55‐70 kDa) was recognized by 5 of 7 sera. However, upon reduction, only 2 of 7 sera from allergic patients reacted with the small subunit (S; 18‐22 kDa; Fag e 1) but also sera from the control group as well as from healthy donors (Figure S4). This raises the question, whether the full‐length protein providing access to all relevant IgE epitopes instead of the small subunit only is useful to clearly identify buckwheat‐allergic patients. Our findings are in agreement with a previous study from Tohgi et al. who compared the reactivity of recombinant Fag e 1 and the purified native legumin. They could discriminate between sensitized but tolerant and allergic patients only by the application of native legumin.^25^ Therefore, we suggest a re‐evaluation whether the full‐length legumin (L‐S; 55‐70 kDa) should be designated Fag e 1. Convincing data that the full‐length buckwheat legumin is an important allergen also emerged from a mouse study, where sensitization of mice with recombinant full‐length legumin resulted in increased expression of Th2 cytokines.^26^

When we tested the individual components, all four allergens showed superior diagnostic specificity as compared to extract‐based buckwheat‐ImmunoCAP. The specificity of Fag e 2 on ImmunoCAP ISAC was relatively high (96%), but showed a diagnostic sensitivity of only 43%. In our ELISA experiments, Fag e 2 showed the best diagnostic specificity (85%) and a sensitivity of 100%. Also others reported that Fag e 2 is associated with immediate hypersensitivity in buckwheat‐allergic patients and is therefore useful for diagnosis.^15^, ^25^

Furthermore, we purified an IgE‐binding protein of about 55 kDa (UniProt accession no. Q6QJL1) with IgE‐binding properties and identified it as a member of the vicilin‐like family of seed storage proteins, designated Fag e 5 by the IUIS allergen nomenclature subcommittee. This is in line with previous studies where vicilins from other food sources such as peanut, walnut, and sesame were identified as important allergens.^27^ Interestingly, in a Korean study, a 19 kDa N‐terminal antimicrobial peptide, cleaved off from a vicilin‐like precursor protein, was identified as major allergen, designated Fag e 3.^17^ In contrast, we did not detect sIgE to a 19 kDa protein within the sera from our cohort. Only our purified mature Fag e 5 was able to bind sIgE. Likewise in other cohorts, binding of sIgE to a 19 kDa component could not be detected.^19^, ^24^

Another protein that turned out to be IgE‐reactive discriminating between sensitized and allergic patients was identified as AMP1/2, antimicrobial peptides belonging to the hevein family.^23^ This newly identified buckwheat allergen was registered in the allergen database (www.allergen.org) and designated Fag e 4. Sequence analysis revealed 65% sequence identity to hevein, a major allergen from *Hevea brasiliensis*. Fag e 4 may be the cross‐reactive allergen that accounts for allergic reaction in patients sensitized to latex upon buckwheat consumption.^28^

Despite a low prevalence of buckwheat allergy, it is one of the major foods causing severe life‐threatening reactions.^3^, ^4^, ^5^, ^6^, ^7^, ^9^ As neither skin testing nor the presence of buckwheat‐sIgE proved to be useful for precisely predicting clinical allergy, the diagnosis is still based on a positive food challenge and/or a clear‐cut history of severe reactions. Interestingly, we found that all patients with buckwheat allergy had a significantly larger SPT weal diameter to buckwheat compared to the positive control, whereas the buckwheat‐sensitized had a significantly smaller SPT weal diameter to buckwheat compared to the positive control. Furthermore, the patient cohort showed that pollen cosensitizations were more common in patients asymptomatically sensitized to buckwheat compared to patients with buckwheat allergy. So far we could not identify a potential candidate for pollen cross‐reaction to buckwheat. The vast majority of patients with buckwheat allergy had no or very few cosensitizations to foods or latex. In contrast, patients asymptomatically sensitized to buckwheat were sensitized to a range of other allergens. This is an interesting finding suggesting that cosensitizations without clinical buckwheat allergy might be primarily induced by other food sources such as peanut, sesame, or hazelnut. In contrast in the allergic group, buckwheat was the sensitizer, and cosensitizations to other food sources were virtually absent. To confirm our findings, it would be interesting to test a larger cohort of buckwheat‐allergic patients as well as patients allergic to other food sources from different regions all over Europe.

Precise in vitro diagnosis of buckwheat allergy as well as a potential prediction of the severity of symptoms is highly needed. While for some food sources (e.g. peanut, hazelnut, kiwifruit) a well‐defined panel of allergens is available for CRD, for buckwheat this is lacking. To date, this is the first study, where several well‐characterized buckwheat allergens were tested simultaneously for their IgE reactivity. Our data indicate that the designation Fag e 1 should be applied to the full‐length protein consisting of both subunits (L‐S; 55‐70 kDa). Furthermore, we confirmed that IgE binding to Fag e 2 is indicative for buckwheat allergy. Additionally, Fag e 4, potentially important for cross‐reactivity to latex was added to the allergen panel of buckwheat. Finally, we characterized a vicilin‐like protein as a new relevant marker allergen, now designated Fag e 5.

## CONFLICT OF INTEREST

The authors declare that they have no conflicts of interest.
