# Supplementary material for: Concomitant sensitization to legumin, Fag e 2 and Fag e 5 predicts buckwheat allergy
Source: Clin Exp Allergy. 2017 Dec 22;48(2):217–24. doi: 10.1111/cea.13068 (PMC5814722; doi:10.1111/cea.13068)
Supplement: Supplementary file 1 [file CEA-48-217-s001.pdf]

Supporting Information

Concomitant sensitization to legumin, Fag e 2 and Fag e 5 predicts buckwheat allergy

S. Geiselhart[1], C. Nagl[1], P. Dubiela[1], A. C. Pedersen[2], M. Bublin[1], C. Radauer[1], C. Bindslev-Jensen[2], K. Hoffmann-Sommergruber[1], and C. G. Mortz[2]

[1]Department of Pathophysiology and Allergy Research, Medical University of Vienna, Vienna, Austria

[2]Department of Dermatology and Allergy Center, ORCA (Odense Research Center for Anaphylaxis), Odense University Hospital, Odense, Denmark

Short title: CRD in buckwheat allergy

Correspondence

Karin Hoffmann-Sommergruber

Department of Pathophysiology and Allergy Research

Medical University of Vienna

Währinger Gürtel 18-20

1090 Vienna, Austria

Tel: +43 1 40400 51040

Fax: +43 1 40400 51300

E-mail: karin.hoffmann@muv.ac.at

Supplementary Methods

Protein extraction and purification of allergens

Raw buckwheat seeds were frozen, finely ground in a blender and proteins were extracted with 4 volumes of extraction buffer (20 mM Tris-HCl, pH 8.0, 3% polyvinyl polypyrrolidone, 10 mM dithiothreitol) at 4°C with stirring overnight. The obtained protein extract was either used for immunoblotting or for purification of allergens.

In a first step, high and low molecular mass proteins were separated by size exclusion chromatography. Concentrated protein extract was dialyzed against column buffer (20 mM Tris-HCl, pH 7.5; 0.3 M NaCl) and loaded onto a HiPrep 26/60 Sephacryl S-200HR column (Thermo Fisher Scientific, Gothenburg, Sweden). The chromatogram showed two major peaks (peak1 and 2). Peak 1, containing the high molecular weight (HMW) proteins, was further separated by anion-exchange chromatography using Q sepharose (Thermo Fisher Scientific) equilibrated with 20 mM Tris-HCl, pH 8.0. Proteins were eluted by a linear gradient of increasing concentration (0–50%) of 1 M NaCl, resulting in three different peaks containing vicilin and legumin. Peak 2, containing the low molecular weight (LMW) proteins, was separated by anion exchange chromatography. The flow through (FT) of the anion exchange column was then further separated by cation exchange chromatography using SP sepharose (Thermo Fisher Scientific) equilibrated with 20 mM ammonium acetate pH 6.5 by a linear gradient of increasing concentration (0-50%) of 1 M NaCl resulting in two main peaks containing AMP1/2.

For the purification of the 2S albumin, size exclusion chromatography was performed as described above. Subsequently, globulins were removed from the low molecular weight (LMW) fraction by precipitation with four volumes of cold methanol (60%, v/v) and prolamins were precipitated with acetone. The prolamin containing fraction was loaded onto a Q Sepharose (Thermo Fisher Scientific) column with 20 mM Tris-HCl, pH 8.0 and proteins bound to the column were eluted with increasing concentrations (0–50%) of 1 M NaCl.

Proteins were detected by their absorbance 280 nm and protein containing fractions were analyzed by SDS-PAGE at reducing and non-reducing conditions.

N-terminal sequencing by Edman degradation

The N-terminal sequence was determined using an Applied Biosystems Procise 491 sequencer (Applied Biosystems, Foster City, CA, USA). Briefly, proteins were separated by SDS-PAGE and blotted to a polyvinylidene difluoride (PVDF) membrane. The proteins were stained with 0.1% (w/v) Coomassie brilliant blue R-250 in 50% (v/v) methanol, 1% (v/v) acetic acid. Bands of interest were excised and ~100 pmol were subjected to sequence analysis.

Protein sequencing by tandem mass spectrometry

Bands of interest were excised manually from Coomassie-stained gels. After washing and destaining bands were reduced with dithiothreitol and alkylated with iodoacetamide.[1] In-gel digestion was performed with trypsin (Trypsin Gold, Mass Spectrometry Grade, Promega, Madison, WI) with a final trypsin concentration of 20 ng/µl in 50 mM aqueous ammonium bicarbonate and 5 mM $CaCl_2$. Digest proceeded for 8 hours at 37°C.[2] Afterwards, peptides were extracted with three changes of 30 µL of 5% trifluoro acidic acid (TFA) in 50% aqueous acetonitrile supported by ultrasonication for 10 min per change. Extracted peptides were dried in a vacuum concentrator (Eppendorf, Hamburg, Germany). Dried peptides were redissolved in 0.1% aqueous TFA prior to LC-MS injection.

Peptides were separated on a nano-HPLC Ultimate 3000 RSLC system (Dionex). Sample pre-concentration and desalting was accomplished with a 5 mm Acclaim PepMap µ-Precolumn (300 µm inner diameter, 5 µm particle size, and 100 Å pore size) (Dionex). For sample loading and desalting 2% acetonitrile in ultra-pure H_2O with 0.05% TFA was used as

a mobile phase with a flow rate of 5 µl/min. For mass spectrometric analysis the LC was directly coupled to a high resolution quadrupole time of flight mass spectrometer (Triple TOF 5600+, Sciex).

For information dependent data acquisition (IDA runs) MS1 spectra were collected in the range of 400-1500 m/z. The 25 most intense precursors with charge state 2–4, which exceeded 100 counts per second, were selected for fragmentation for 250 ms. MS2 spectra were collected in the range 100–1800 m/z for 110 ms. Precursor ions were dynamically excluded from reselection for 12 s. The nano-HPLC system was operated by Chromeleon 6.8 (Dionex) and the MS by Analyst Software 1.6 (Sciex).

Spectra were searched in SwissProt or NCBI DB (downloaded from the publicly available servers (http://www.ncbi.nlm.nih.gov/, www.uniprot.org).

References

1. Shevchenko A, Wilm M, Vorm O, Mann M. Mass spectrometric sequencing of proteins silver-stained polyacrylamide gels. *Anal Chem* 1996;**68**:850–858.

2. Jiménez CR, Huang L, Qiu Y, Burlingame AL, Jiménez CR, Huang L et al. In-Gel Digestion of Proteins for MALDI-MS Fingerprint Mapping. In: *Current Protocols in Protein Science*. 1998: 16.4.1-16.4.5.

Supplementary Figures and Tables

Study population

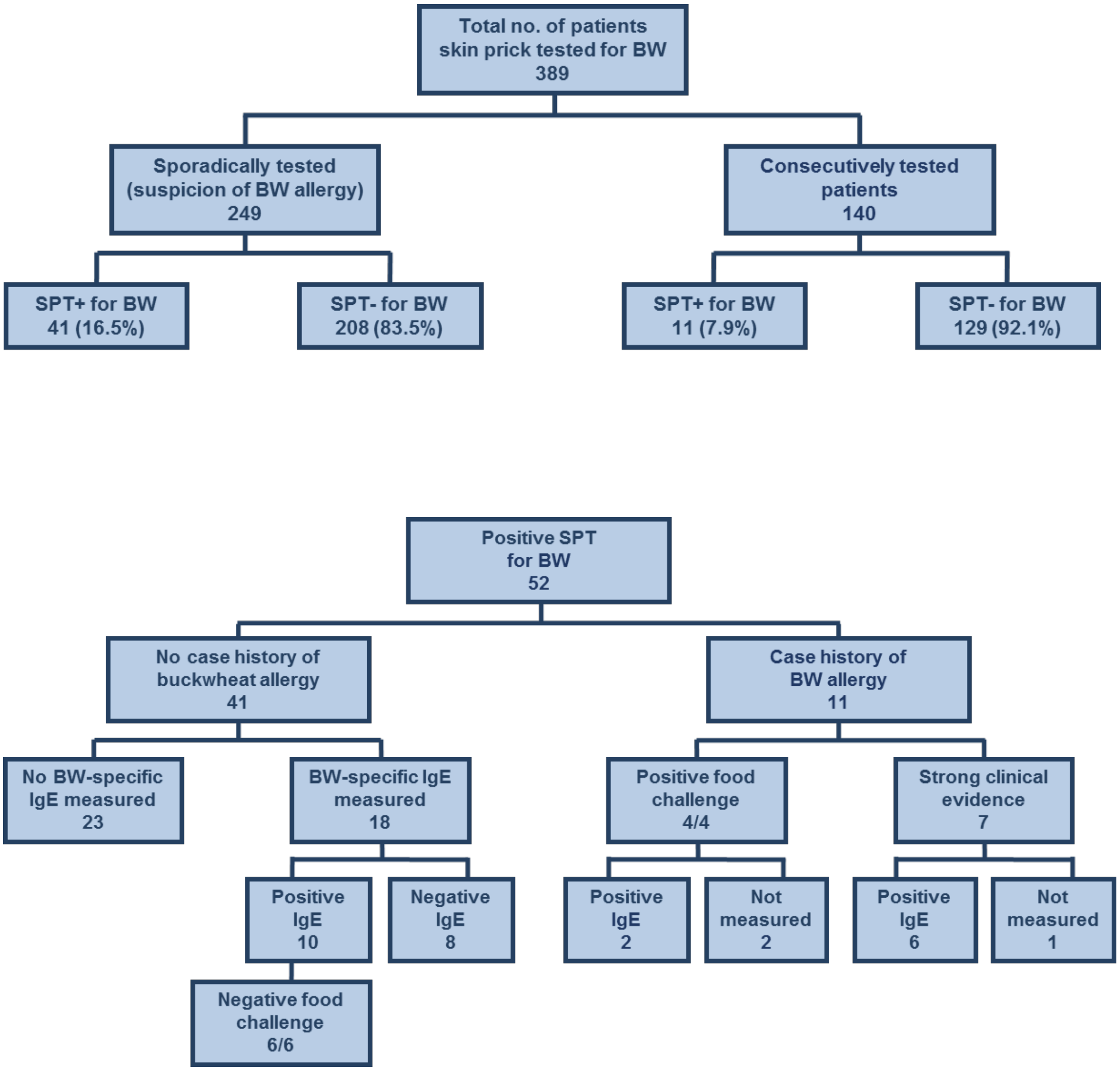

Figure S1 Results of buckwheat skin prick tests and diagnosis of buckwheat allergy in skin prick test positive patients

Purification strategy

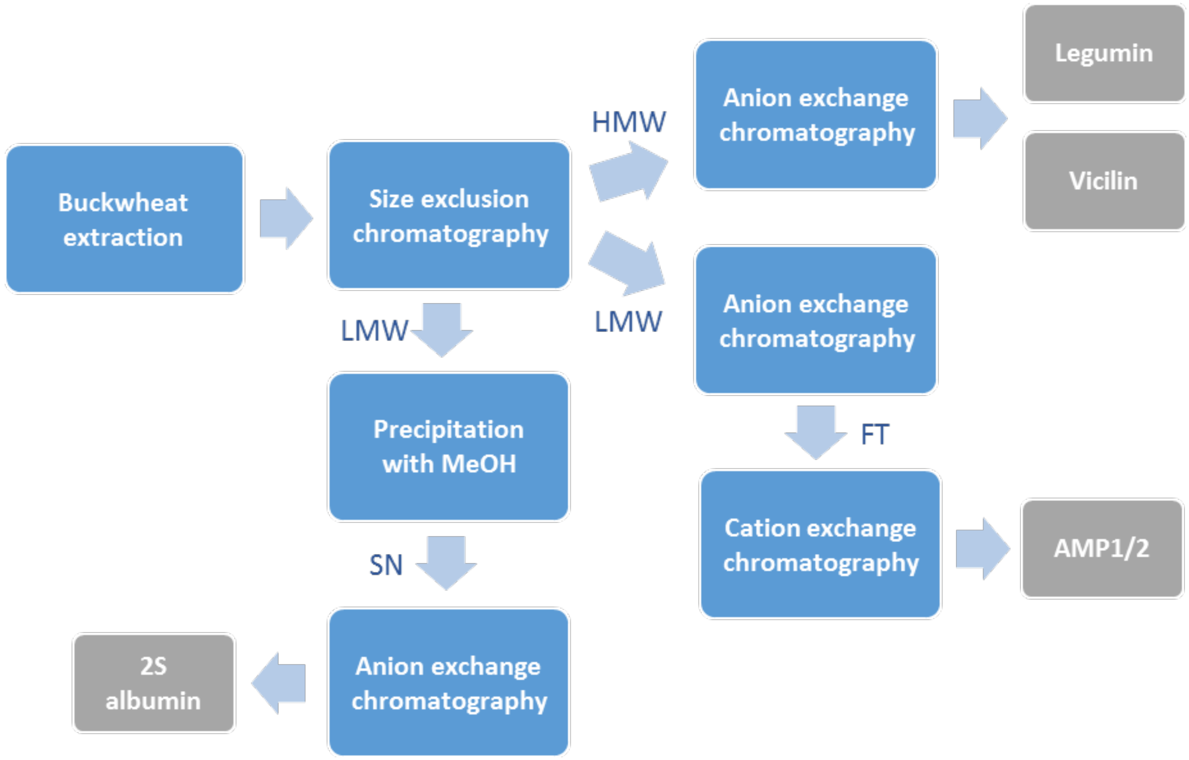

Figure S2 Flow chart of the different purification steps. HMW, high molecular weight proteins; LMW, low molecular weight proteins; FT flow through; SN, supernatant

Identification of the purified proteins

Fag e 4 (AMP1/2)

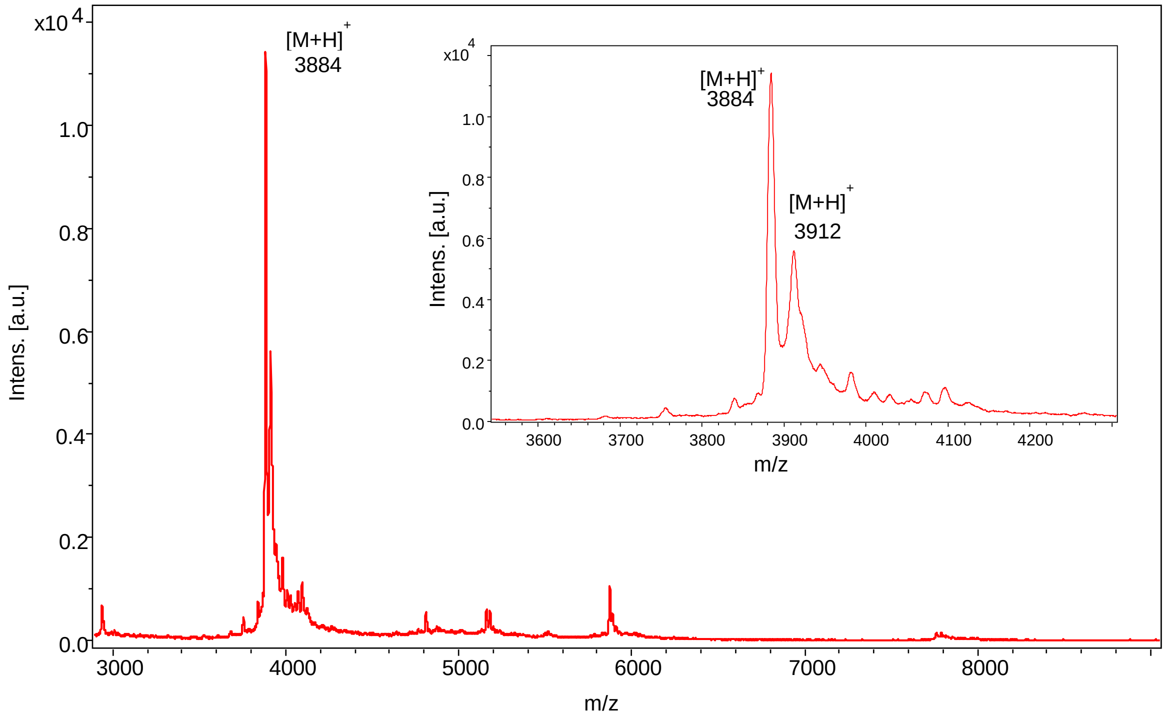

AMP1 (Theoretical mass: 3879 Da)

P0DKH7|AMP1 Antimicrobial peptide 1 *Fagopyrum esculentum*
AQCGAQGGGATCPGGLCCSQWGWCGSTPKYCGAGCQSNCK

AMP2 (Theoretical mass: 3907 Da)

P0DKH8|AMP2 Antimicrobial peptide 2 *Fagopyrum esculentum*
AQCGAQGGGATCPGGLCCSQWGWCGSTPKYCGAGCQSNCR

Figure S3A MALDI-TOF MS spectra of Fag e 4 (AMP1/2) and sequences with calculated theoretical molecular masses. The underlined sequences were confirmed by N-terminal sequencing.

Fag e 2 (2S albumin)

Q2PS07|16 kDa allergen *Fagopyrum esculentum*
MKLFIILATATLLIAATQATYPRDEGFDLGETQMSSKCMRQVKMNEPHLKKCNRYIAMDI
LDDKYAEALSRVEGEGCKSEESCMRGCCVAMKEMDDECVCEWMKMMVENQKGRIGERLIK
EGVRDLKELPSKCGLSELECGSRGNRYFV

Figure S3B Full-length sequence of buckwheat 2S albumin. Signal peptide in bold letters; the underlined sequence was confirmed by N-terminal sequencing

Legumin

BAO50858.1 13S globulin *Fagopyrum esculentum*
MSTKLILSFSLCLMVLSCSAQLLPWQKGQRSRPHHGHQQFQHQCDVQRLTASEPSRRVRSEAGVTEIWDN
DTPEFRCAGFVAVRVVIQPGGLLLPSYSNAPYITFVEQGRGVQGVVVPGCPETFQSESEFEYPQSQRDQR
SRQSESEESSRGDQRTRQSESEEFSRGDQHSRQSESEESSHGDQHQKIFRIRDGDVIPSPAGVVQWTHND
GDNDLISITLYDANSFQNQLDGNVRNFFLAGQSKQSREDRRSQRQTREEGGDRQSRESDDDEALLEANIL
SGFQDEILQEIFRNVDQETISKLRGETDQRGFIVQARDLKLRVPEEYEEELQRERGDRKRGGSGRSNGLE
QAFCNLKFKQNVNRPSRADVFNPRAGRINTVNSNNLPILEFIQLSAQHVVLYKNAILGPRWNLNAHSALY
VTRGEGRVQVVGDEGRSVFDDNVQRGQILVVPQGFAVVLKAGNEGLEWVELKNDDNAITSPIAGKTSVLR
AIPVEVLANSYDISTKEAFRLKNGRQEVEVFRPFQSRDEKERERYSIV

Figure S3C Full-length sequence of buckwheat legumin. Signal peptide in bold letters; the small subunit (Fag e 1) is marked in grey; the underlined sequences sere confirmed by tandem mass spectrometry

Fag e 5 (vicilin-like)

AAS48514.1 vicilin-like protein, partial *Fagopyrum esculentum*
IAVVTNGKGKFQMACPHISAEGKQSKRQREGKSIVHYETVNGDLSSGTVFVVPAGHPFVTAASLEDNLEL
ICFEVNADDNERIPLAGKNSLFKQFEREAKELAFEEKADVVDKLLEKQQQEFFFEGPRRRKEQEAGRSDA

Figure S3D Partial sequence of buckwheat Fag e 5. The underlined sequences were confirmed by tandem mass spectrometry

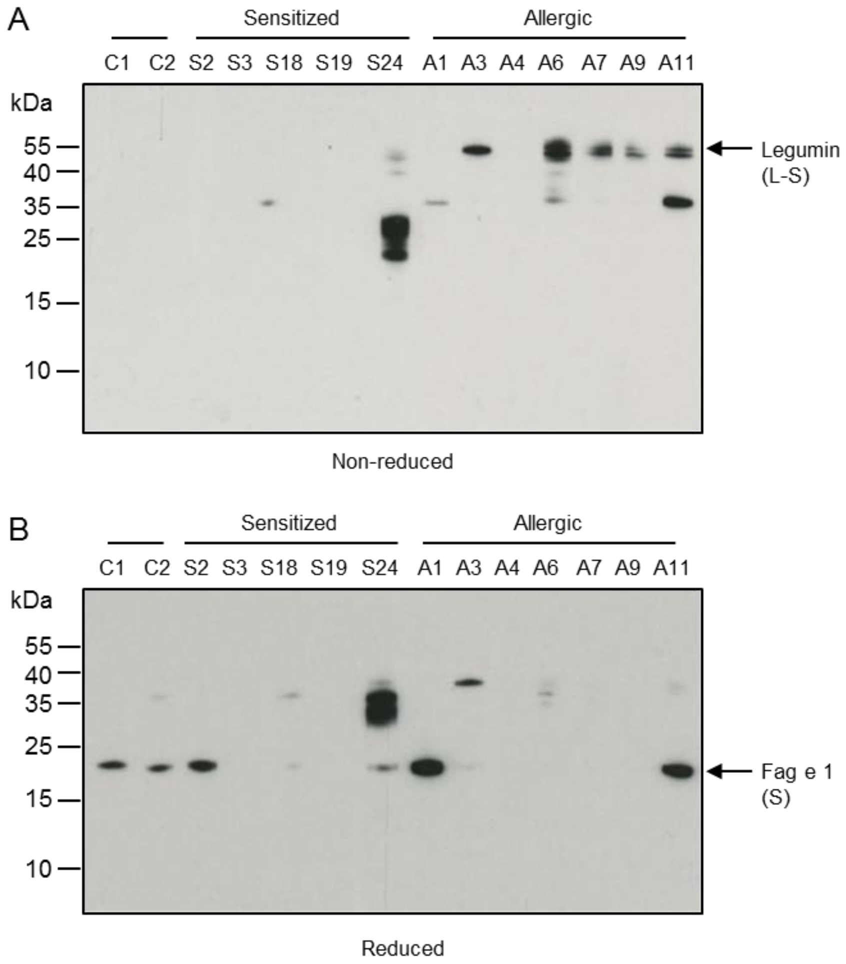

Figure S4 IgE immunoblot of buckwheat legumin at non-reducing (A) and reducing (B) conditions with sera from buckwheat-sensitized and buckwheat-allergic patients. Sera from healthy donors served as controls (C1, C2). Fag e 1, small subunit (S) of legumin; disulphide-linked large and small subunit (L-S) of legumin. Molecular masses (kDa) are indicated on the left.

TABLE S1 Characteristics of patients with buckwheat allergy

	**Sex (F/M)<br>Age (years)**	**SPT BW<br>Wheal diam.<br>(mm)**	**SPT Histamine<br>Wheal diam.<br>(mm)**	**BW-specif.<br>IgE level (kU_A/L)<br>HR class**	**Reaction to BW in food<br>Grade of anaphylaxis (according to Sampson)**
**Patient A1**	F, 66	10	7	nd<br>3	**Grade 5**<br>Skin: flushing, angioedema<br>GI-tract: nausea, vomiting, diarrhea<br>Respiratory tract: coughing<br>Cardiovascular: hypotension<br>Neurological: loss of consciousness
**Patient A2**	F, 43	9	5	nd<br>4	**Grade 2**<br>Skin: angioedema, urticaria
**Patient A3**	F, 53	8	4.5	11.6<br>nd	**Grade 4**<br>Skin: angioedema<br>Respiratory tract: dyspnea
**Patient A4**	F, 34	9.5	6	1.9<br>0	**Grade 4**<br>Skin: itching in the palms, angioedema<br>Respiratory tract: dyspnea, a burning sensation in the throat<br>Cardiovascular: hypotension
**Patient A5**	F, 67	7.5	5.5	nd<br>3	**Grade 3**<br>Skin: itching in the palms<br>GI-tract: diarrhea, nausea
**Patient A6**	F, 44	9	7	6.6<br>3	**Grade 4**<br>Respiratory tract: dyspnea, asthma<br>Neurological: dazed
**Patient A7**	F, 55	25.5	8.5	4.5<br>3	**Grade 4**<br>Skin: itching in the scalp, palms and soles, generalized urticarialangioedema<br>GI-tract: itching in the mouth, vomiting<br>Respiratory tract: dyspnea
**Patient A8**	F, 60	10	5	36.9<br>3	**Grade 4**<br>Skin: angioedema, urticaria<br>GI-tract: vomiting, diarrhea
**Patient A9**	F, 54	10	5.5	3<br>nd	**Grade 4**<br>Skin: universal itching, angioedema, urticaria<br>GI-tract: itching in the mouth, vomiting, diarrhea, epigastric pain<br>Respiratory tract: dyspnea<br>Cardiovascular: hypotension
**Patient A10**	F, 50	12	7.5	2.3<br>nd	**Grade 4**<br>Skin: urticaria, angioedema<br>Respiratory tract: dyspnea, asthma
**Patient A11**	F, 46	12	7.5	4.8<br>nd	**Grade 3**<br>Skin: angioedema<br>GI-tract: itching in the mouth<br>Respiratory tract: sensation of throat pruritus or tightness

Abbreviations: BW, buckwheat; SPT, skin prick test; HR, histamine release; nd, not determined; Patient A1-A2 positive DBPCFC, patient A3 and A11 positive OFC, Patient A4-A10: strong clinical evidence for buckwheat allergy.

TABLE S2 Sensitization patterns (positive SPT) among buckwheat sensitized and allergic patients

Allergen source	BW-sensitized (n=41)			BW-allergic (n=11)		
	SPT+, n (%)	SPT-, n (%)	nd, n	SPT+, n (%)	SPT-, n (%)	nd, n
**Pollen**	32 (80%)	8 (20%)	1	5 (50%)	5 (50%)	1
Birch	18 (47%)	20 (53%)	3	1 (10%)	9 (90%)	1
Grass	28 (70%)	12 (30%)	1	4 (40%)	6 (60%)	1
Mugwort	15 (39%)	23 (61%)	3	2 (22%)	7 (78%)	2
**Hazelnut**	26 (70%)	11 (30%)	4	2 (25%)	6 (75%)	3
**Wheat**	28 (68%)	13 (32%)	0	0 (0%)	9 (100%)	2
**Cereal grains**	20 (71%)	8 (29%)	13	0 (0%)	7 (100%)	4
**Sesame**	19 (53%)	17 (47%)	5	0 (0%)	6 (100%)	5
**Soy**	17 (46%)	20 (54%)	4	0 (0%)	8 (100%)	3
**Poppy seed**	15 (42%)	21 (58%)	5	0 (0%)	6 (100%)	5
**Peanut**	14 (38%)	23 (62%)	4	0 (0%)	8 (100%)	3
**Latex**	4 (12.5%)	28 (87.5%)	9	0 (0%)	8 (100%)	3

Abbreviations: BW, buckwheat; SPT, skin prick test; n, number; nd, not determined; cereal grains = barley, oats or rye; pollen = grass, birch or mugwort.

TABLE S3 IgE sensitization among buckwheat SPT positive patients

	**BW-sensitized IgE BW+ n=10**			**BW-sensitized IgE BW- n=8**			**BW-allergic* n=11**		
**Allergen source**	**IgE+ n (%)**	**IgE- n**	**ND n**	**IgE+ n (%)**	**IgE- n**	**ND n**	**IgE+ n (%)**	**IgE- n**	**ND n**
**Pollen**	9 (90%)	1	0	3 (43%)	4	1	4 (36%)	7	0
Birch	6 (67%)	3	1	3 (50%)	3	2	0	10	1
Grass	9 (90%)	1	0	1 (17%)	5	2	4 (36%)	7	0
Mugwort	6 (67%)	3	1	1 (17%)	6	1	1 (13%)	7	3
**Hazelnut**	6 (100%)	0	4	0	1	7	1 (25%)	3	7
**Wheat**	6 (100%)	0	4	3 (50%)	3	2	1 (14%)	7	3
**Cereal grains**	5 (100%)	0	5	2 (40%)	3	3	0	6	5
**Sesame**	5 (100%)	0	5	1	0	7	0	2	9
**Soy**	7 (100%)	0	3	0	1	7	1	0	10
**Poppy seed**	3 (75%)	1	6	0	1	7	0	2	9
**Peanut**	6 (100%)	0	4	0	2	6	1	0	10
**Latex**	1	0	9	0	2	6	1 (33%)	2	8

Abbreviations: BW, buckwheat; cereal grains = barley, oats or rye; pollen = grass, birch or mugwort.

* In 3/11 with buckwheat allergy no measurement of specific IgE to buckwheat was performed but the 3 had a positive HR test to buckwheat class 3-4.

TABLE S4 IgE reactivity to individual components tested using the ImmunoCAP ISAC microarray

	BW-sensitized (n=27)		BW-allergic (n=7)	
	**n**	**Median (range)**	**n**	**Median (range)**
**Storage proteins**				
Ana o 2	4	2.95 (0.5-17)	-	-
Cor a 9	2	4.25 (1.7-6.8)	-	-
Jug r 1	1	0.80 (0.8-0.8)	-	-
Jug r 2	4	0.85 (0.4-12)	-	-
Fag e 2	1	0.80 (0.8-0.8)	3	3.8 (2.6-10)
Tri a aA_Ti	1	2.80 (2.8-2.8)	-	-
Ara h 1	2	32.00 (17-47)	-	-
Ara h 2	3	15.90 (0.9-100)	-	-
Ara h 3	2	53.70 (7.4-100)	-	-
Ara h 6	3	18.50 (1-100)	-	-
Gly m 5	3	0.80 (0.4-7.9)	-	-
Gly m 6	3	17.00 (0.3-100)	-	-
Ses I 1	1	24.00 (24-24)	-	-
**PR-10**				
Bet v 1	10	75.00 (1.1-100)	-	-
Aln g 1	9	7.70 (0.6-57)	-	-
Cor a 1.0101	8	2.10 (0.8-35)	-	-
Cor a 1.0401	10	7.85 (1.2-44)	-	-
Mal d 1	9	17.00 (9.8-34)	-	-
Pru p 1	9	9.80 (0.7-21)	-	-
Gly m 4	6	4.80 (1.1-14)	-	-
Ara h 8	9	1.60 (0.7-28)	-	-
Act d 8	3	1.50 (1.3-9.4)	-	-
Api g 1	1	2.50 (2.5-2.5)	-	-
**Profilin**				
Bet v 2	7	1.40 (0.4-2.6)	-	-
Hev b 8	6	6.90 (0.9-13)	-	-
Mer a 1	7	3.80 (0.4-8.8)	-	-
Phl p 12	5	1.10 (0.5-1.3)	-	-
**nsLTP**				
Ara h 9	8	2.35 (0.3-11)	-	-
Cor a 8	6	1.05 (0.4-14)	-	-
Jug r 3	7	2.80 (1-11)	-	-
Pru p 3	8	2.70 (0.8-7.4)	-	-
Art v 3	7	1.60 (0.4-8.4)	-	-
Ole e 7	2	1.70 (0.6-2.8)	-	-
Pla a 3	8	1.90 (0.5-4.8)	-	-
Par j 2	1	7.20 (7.2-7.2)	-	-
Tri a 14	3	9.50 (0.4-1.1)	-	-

Abbreviations: BW, buckwheat.
